# Targeted blockade in lethal West Nile virus encephalitis indicates a crucial role for very late antigen (VLA)-4-dependent recruitment of nitric oxide-producing macrophages

**DOI:** 10.1186/1742-2094-9-246

**Published:** 2012-10-30

**Authors:** Daniel R Getts, Rachael L Terry, Meghann Teague Getts, Marcus Müller, Sabita Rana, Celine Deffrasnes, Thomas Myles Ashhurst, Jane Radford, Markus Hofer, Shane Thomas, Iain L Campbell, Nicholas JC King

**Affiliations:** 1 The Discipline of Pathology, School of Medical Sciences Faculty of Medicine, The University of Sydney, Blackburn Building D06, Sydney, NSW 2006, Australia; 2 The School of Molecular Biosciences, Faculty of Science, University of Sydney, Sydney, Australia; 3 The Bosch Institute, University of Sydney, Sydney, Australia; 4 The Centre for Vascular Research, School of Medical Sciences, University of New South Wales, Sydney, Australia

**Keywords:** Neurotropic virus, Flavivirus, Inflammatory monocytes, West Nile virus encephalitis, Macrophage infiltration, VLA-4, Integrins, Nitric oxide

## Abstract

Infiltration of Ly6C^hi^ monocytes from the blood is a hallmark of viral encephalitis. In mice with lethal encephalitis caused by West Nile virus (WNV), an emerging neurotropic flavivirus, inhibition of Ly6C^hi^ monocyte trafficking into the brain by anti-very late antigen (VLA)-4 integrin antibody blockade at the time of first weight loss and leukocyte influx resulted in long-term survival of up to 60% of infected mice, with subsequent sterilizing immunity. This treatment had no effect on viral titers but appeared to be due to inhibition of Ly6C^hi^ macrophage immigration. Although macrophages isolated from the infected brain induced WNV-specific CD4^+^ T-cell proliferation, T cells did not directly contribute to pathology, but are likely to be important in viral control, as antibody-mediated T-cell depletion could not reproduce the therapeutic benefit of anti-VLA-4. Instead, 70% of infiltrating inflammatory monocyte-derived macrophages were found to be making nitric oxide (NO). Furthermore, aminoguanidine-mediated inhibition of induced NO synthase activity in infiltrating macrophages significantly prolonged survival, indicating involvement of NO in the immunopathology. These data show for the first time the therapeutic effects of temporally targeting pathogenic NO-producing macrophages during neurotropic viral encephalitis.

## Findings

During autoimmune or infectious inflammation of the central nervous system (CNS), inflammatory monocytes migrate into the brain, where they can differentiate into microglia, macrophages, and even dendritic cells [1-3]. The major function of infiltrating inflammatory monocytes is to initiate and amplify the immune response [4-6], but we have recently shown that immigration of these cells into the brain infected with West Nile virus (WNV) contributes directly to the immune pathology of the disease [2]. Inhibition of migration of these cells by anti-chemokine (C-C motif) ligand 2 (CCL2) antibody resulted in a short prolongation of survival in WNV-infected animals [2]. In this report, we extend these findings to show that inflammatory monocyte-derived macrophages not only migrate into the WNV-infected CNS, utilizing very late antigen (VLA)-4, but play a significant role in driving the immune-mediated pathology, at least in part through sustained production of NO in the symptomatic stages of disease. Importantly, for potential clinical translation, we show that pulsed anti-VLA-4 therapy during acute disease increases the survival of infected animals.

## Methods

### Ethics approval

All procedures were conducted with approval from the animal ethics committee of Sydney University.

### Animals

Female C57BL/6 mice were intranasally inoculated with 6 × 10^4^ or 6 × 10^3^ plaque-forming units (PFU) WNV (Sarafend; Generated as described in 2) or phosphate buffered saline (PBS; Sham control) under anesthesia at 8 weeks old, as previously described [2,7]. For *in vivo* antibody treatments, mice were injected daily with 100 μg anti-CD49d (VLA-4), CD11a (lymphocyte function-associated antigen-1; LFA-1), CD4, CD8 or isotype control antibodies (Biolegend, San Diego, CA, USA intraperitoneally from day 6 post-infection (p.i.) titrated for maximal effect. For *in vivo* NO inhibition, mice were injected with 300 mg/kg aminoguanidine hemisulfate (Sigma) intraperitoneally from day 6 p.i. For *in vivo* cathespin labeling, mice were intravenously injected with 2 nmol/l of a fluorescent agent (ProSense; VisEn Medical, Bedford, MA, USA) on day 6 p.i. Mice were killed on day 7 p.i. by cardiac perfusion under anesthesia, as previously described [7]. To determine virus titers, plaque assays using homogenized brain on baby hamster kidney cell monolayers were conducted as previously described [7]. For immunohistology, brains were cut into 8 μm sections and stained with anti-CD54 (intercellular adhesion molecule-1; ICAM-1), CD106 (vascular cell adhesion molecule 1; VCAM-1), isotype control antibodies (Biolegend), or anti-iNOS (Neomarkers Inc., Fremont, CA, USA), using a previously described immunohistochemical protocol [2]. *In situ* hybridization for NOS2 was conducted as previously described [7], using a P33-labeled cRNA probe. For flow-cytometry analysis, leukocytes isolated from the brain and blood were incubated with CD16/32 antibody and then labeled with fluorochrome-conjugated CD45, CD11b, Ly6C, Ly6G, F4/80, CD3, CD4, CD8, major histocompatibility complex (MHC)-II, CD80, CD86, CD49d (VLA-4), CD11a (LFA-1) or isotype control antibodies (Biolegend), using a previously described flow-cytometry protocol [2]. For detection of intracellular NO, cells were incubated with 5 μmol/l of a green fluorescent marker that is activated by NO (4-amino-5-methylamino-2',7'-difluorofluorescein diacetate (DAF-FM); Invitrogen Corp., Carlsbad, CA, USA) for 30 minutes as previously described [8]. Fluorescence was measured, and cell sorting carried out (FACS ARIA; Becton Dickinson, Franklin Lakes, NJ, USA) and analyzed using flow-cytometry software (FlowJo; TreeStar Inc., Ashland, OR, USA). For T-cell proliferation assays, Ly6C^hi^ macrophages, Ly6C^−^ microglia and CD4^+^ T cells were sorted from the brains of colony-stimulating factor 1 receptor (cFMS)-enhanced green fluorescent protein (EGFP) chimeras (described previously [2]) and spleens of C57BL/6 mice on day 7 p.i. cFMS-EGFP is the promoter for CD115 (the macrophage colony-stimulating factor receptor), thus, in these mice, macrophages express EGFP. Assays using 3-(4,5-dimethylthiazol-2-yl)-2,5-diphenyltetrazolium bromide (MTT) assays were conducted in accordance with the manufacturer’s instructions (Invitrogen Corp.), with cells plated at the indicated ratios and incubated for a total of 72 hours.

### Statistical analysis

Statistical analysis was conducted using one-way ANOVA with a Tukey-Kramer *post hoc* test, and statistical analysis of survival was conducted using the log-rank (Mantel-Cox) test. Data shown are means ± standard deviations. *P *< 0.05 was considered significant.

## Results and discussion

### Expression of integrins and cellular adhesion molecules

**Figure 1:**
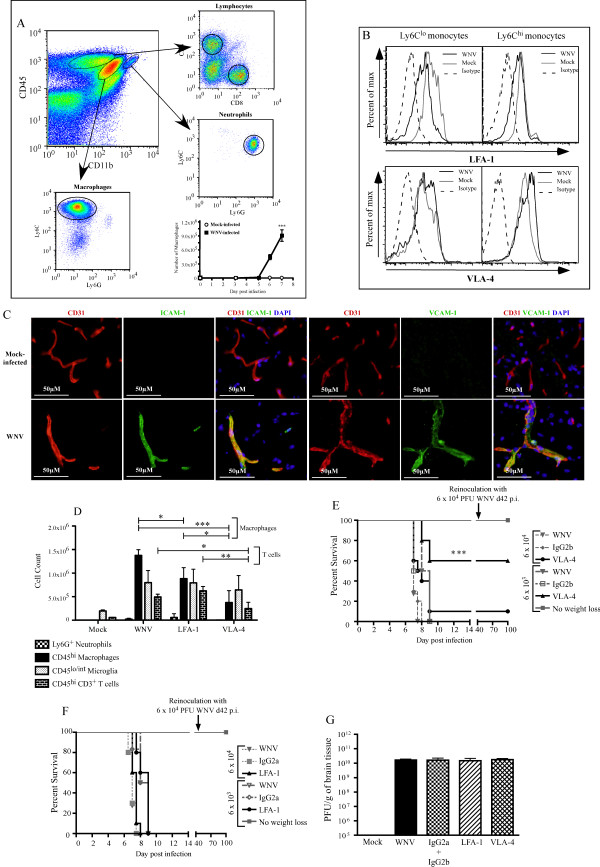
**VLA-4 is the primary integrin for inflammatory monocyte derived macrophages.** Analysis at day 7 p.i. showed (**A**) CD4^+^ and CD8^+^ T cells, Ly6G^+^ neutrophils (~6%, 7% and 3%, respectively, of all infiltrating leukocytes), and large numbers of Ly6C^+^ macrophages (~50% of infiltrating leukocytes, of which >90% were Ly6C^hi^) infiltrating into the brain from day 5 p.i., and (**B**) lymphocyte function-associated antigen (LFA)-1 and very late antigen (VLA)-4 was expressed on Ly6C^lo^ and Ly6C^hi^ blood monocytes. (**C**) Immunohistochemical staining for intercellular adhesion molecule (ICAM)-1 and vascular cell adhesion molecule (VCAM)-1 performed on day 7 p.i. on the sham-infected and WNV-infected brains of C57BL/6 mice showed significant widespread upregulation of both molecules in WNV-infected mice and co-localized with CD31. Treatment with 100 μg anti-VLA-4 antibody from day 6 p.i. onwards resulted in reduced neutrophil and T cell recruitment into day 7 p.i. WNV-infected brains. A 66% reduction was seen in the CD45^+^/CD11b^+^/Ly6G^−^/Ly6C^hi^ macrophage compartment. (**D**) By contrast, LFA-1 blockade reduced Ly6C^hi^ macrophage infiltration by 33%. (**E**) VLA-4 blockade resulted in increased survival of 10 to 60%, depending on the inoculation dose (*P *< 0.001), whereas (**F**) LFA-1 blockade did not increase survival. (**G**) Viral titers in the brain at day 7 p.i. were similar in all treatment groups. Data are representative of at least three independent experiments with at least four mice per group. Antibody-mediated effects on the disease course were determined on four separate occasions with at least ten mice per group. Data shown are the mean ± standard deviation. Statistical analysis was conducted using one-way ANOVA with a Tukey-Kramer *post hoc* test. Statistical analysis of survival was conducted using the log-rank (Mantel-Cox) test. * *P *< 0.05; **P < 0.001; ***P < 0.001. The inoculation dose used was 6 × 10^3^ plaque-forming units, unless otherwise noted.

Previously, we have shown that WNV infection triggers significant limbic-seizure responses that occur immediately preceding death [7]. In addition, acute weight loss correlates with the infiltration of T cells (14%) and neutrophils (3%), but the most striking infiltration (by both number and percentage) are Ly6C-expressing inflammatory macrophages (~50%) (Figure 1A). This infiltration occurs between days 5 and 7 p.i. (Figure 1A) and without detectable blood–brain barrier disruption in most mice [2]. We could not isolate any from sham-infected mice (Figure 1A) [2]. The mechanism by which inflammatory monocytes enter the brain during WNV infection has not been addressed. LFA-1 and VLA-4 are integrins important for trans-endothelial migration [9], and were expressed on blood monocytes in WNV-infected and sham-infected mice (Figure 1B). Although a trend toward increased VLA-4 expression on monocytes from WNV-infected mice was seen, it was not significant. Furthermore, expression of the respective ligands for these integrins, ICAM-1 and VCAM-1, was upregulated in the WNV-infected brain. Expression of both ligands was found on platelet endothelial cell adhesion molecule-positive cerebrovascular endothelium (Figure 1C). To investigate the functional role of these integrins, we injected VLA-4-blocking antibody intraperitoneally into WNV-infected mice on day 6 p.i. Although T-cell numbers decreased, the most dramatic reduction was seen in inflammatory monocyte-derived Ly6C^hi^ macrophages. This reduction was significantly greater in mice treated with anti-VLA-4 than in those treated with anti-LFA-1 (Figure 1D). VLA-4 antibody blockade resulted in reduced encephalitic symptoms, including a reduction in the incidence of seizures (not shown), culminating in enhanced long-term survival of 10 to 60% of mice (*P *< 0.001), with subsequent immunity to virus rechallenge, depending on the inoculating dose of virus used (Figure 1E). By contrast, LFA-1 blockade had no effect on the disease course (Figure 1F). Neutralization of LFA-1 or VLA-4 had no effect on viral titers in the brain on day 7 p.i. (Figure 1G), suggesting that survival is not the result of reductions in viral burden or virus cytotoxicity. However, the data support our previous findings suggesting that increased survival is the direct result of decreased inflammatory monocyte trafficking into the brain [2]. 

**Figure 2:**
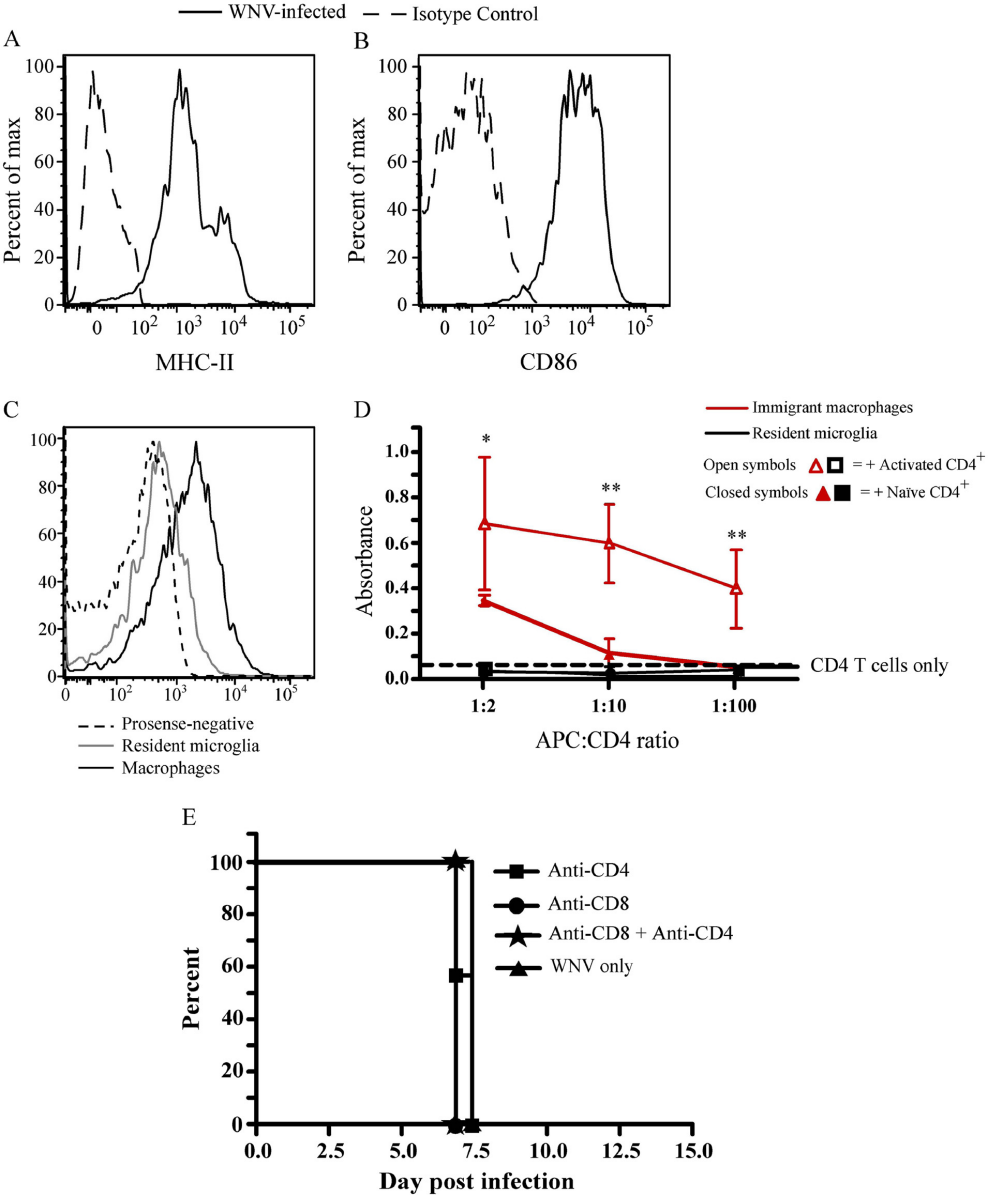
**Assessment of macrophages isolated from the West Nile virus (WNV) infected brain. **(**A**, **B**) Macrophages isolated from day 7 p.i. brain tissue infected with West Nile virus expressed major histocompatability complex (MHC)-II and CD86. (**C**) More than 70% of Ly6C^hi^ macrophages in the WNV-infected brain were positive for fluorescent agent (ProSense; VisEn Medical, Bedford, MA, USA), indicative of cathepsin activity. (**D**) The ability of macrophages (CD45^+^/CD11b^+^/Ly6G^−^/GFP^+^/CD11c^−^/Ly6C^hi^) and resident microglia (CD45^lo/int^/CD11b^+^/GFP^−^/CD11c^−^/Ly6C^hi^), isolated from WNV-infected colony-stimulating factor 1 receptor (cFMS)-enhanced green fluorescent protein (EGFP) chimeric mice [2] to stimulate naïve and activated CD3^+^/CD4^+^ T cells from sham-infected and WNV-infected mice, respectively, was measured. Except at the highest antigen-presenting cell (APC):CD4 ratios, neither population was capable of stimulating naive T-cell proliferation, as measured by 3-(4,5-dimethylthiazol-2-yl)-2,5-diphenyltetrazolium bromide (MTT) assay. (**E**) Notwithstanding the apparent APC capabilities, depletion of CD8^+^ or CD4^+^ or CD4^+^ and CD8^+^ had no effect on overall survival in this model. All experiments were performed at least twice with four to five mice per group. Cells were sorted from 10 pooled cFMS-EGFP chimeric mice, and data represent the mean of triplicate wells and the standard deviation of absorbance. T-cell depletion was performed at least twice with eight to ten mice per group. Statistical analysis was conducted using one-way ANOVA with a Tukey-Kramer *post hoc* test. * Indicates significance of comparison of CD4^+^ T-cell proliferation between wells with macrophages and wells with CD4^+^ T cells only. * *P *< 0.05; **P < 0.001; ***P < 0.001. Mice infected with 6 ×10^4^ plaque-forming units were used to obtain APC populations.

The mechanism by which these cells contribute to the pathogenesis of disease is unresolved. To address this, we initially investigated the ability of these cells to process and present antigen to T cells, as CNS macrophages exhibited cathepsin activity and expressed MHC-II and CD86 (Figure 2A-C), and we hypothesized that infiltrating macrophages might exert their pathogenic effects indirectly acting as antigen presenting cells (APC), via T-cell stimulation. Using naive and activated CD4^+^ T cells, we tested from day 7 p.i. the capacity of immigrant brain macrophages and resident microglia to stimulate T-cell proliferation (Figure 2D). Whereas the control Ly6C^–^ (resident) microglia failed to stimulate T-cell proliferation, inflammatory monocyte-derived macrophages were capable of inducing proliferation of activated T cells, supporting previous studies showing that macrophages can stimulate anti-WNV T-cell responses [10]. It is tempting to speculate from these data that inflammatory monocyte-derived macrophages may cause immunopathology via their influence on T cells [11]; however, deletion of either CD4^+^ or CD8^+^ T cells, or both, did not increase survival time in these mice (Figure 2E). When considered with the reduced number of T cells seen after anti-VLA-4 treatment, these data potentially argue against a role for T cells in causing the observed immune pathology in WNV-infected mice. Indeed, previous studies have shown that in concert with neuronal intracellular antiviral defenses, T cells are important for viral clearance [12-14]. Examination of these pathways in VLA-4 treated mice will be needed to clarify these viral eradication mechanisms precisely. 

**Figure 3:**
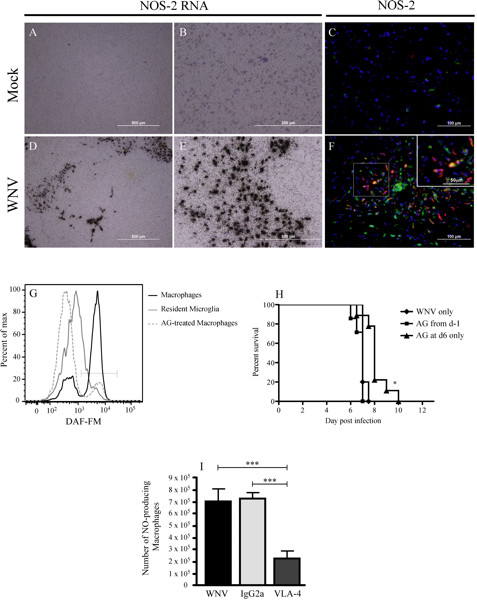
**Macrophages produce NO, which is pathogenic during WNV infection. **(**A**-**E**) Analysis of sections from sham-infected and West Nile virus (WNV)-infected mice at day 7 p.i. showed significant upregulation of nitric oxide synthase (NOS)-2 message in the WNV-infected brains. (**C**, **F**) Furthermore, immunohistochemical staining for NOS-2 protein (red) and lectin (green) showed that most cells expressing NOS-2 in the WNV-infected brain parenchyma at day 7 p.i. were lectin-positive. (**G**) Using the dye 4-amino-5-methylamino-2',7'-difluorofluorescein diacetate (DAF-FM), which, in the presence of NO becomes fluorescent, flow cytometry showed that more than 70% of macrophages (CD45^hi^/CD11b^+^/CD11c^−^/Ly6C^+^) expressed NO compared with approximately 25% of resident microglia (CD45^lo/int^/CD11b^+^/CD11c^−^/Ly6C^−^), and treatment of mice with aminoguanidine resulted in a significant reduction in macrophages expressing NO. (**H**) Inhibition of NO with aminoguanidine did not increase survival when used over the entire infectious period, but it had a demonstrably protective effect given at day 6 p.i. (*P *< 0.05). (**I**) A significant reduction in DAF-FM-positive macrophages was seen from day 7 p.i. in the brains of WNV-infected mice that had been treated with anti-VLA-4 neutralizing antibodies on day 6 p.i. *In situ* hybridization was performed on sagittal brain sections from at least six mice per group. DAF-FM data are representative of three experiments with at least three mice per group. Aminoguanidine therapy was performed four times with at least ten mice per group. Values shown are means ± SD. Statistical analysis was conducted using one-way ANOVA with Tukey-Kramer *post hoc* test. * *P *< 0.05; **P < 0.001; ***P < 0.001. Statistical analysis of survival was determined using the log-rank (Mantel-Cox) test. *P < 0.05 was considered significant.

Because NO production during viral infection has been shown to be protective in some instances, and pathogenic in others, we investigated the possible pathogenic role of NO production by macrophages [15-18]. *In situ* hybridization and immunohistochemistry for inducible NO synthase (NOS)-2 showed significant expression in the WNV-infected brain at day 7 p.i. (Figure 3A-F). Flow cytometry using the NO marker DAF-FM diacetate [8], which becomes fluorescent when it reacts with NO, showed that 72% of macrophages in the WNV-infected brain expressed NO, compared with only 24% of resident microglia (CD45^Lo^/Ly6C^−^) (Figure 3G). Furthermore, intraperitoneal administration of aminoguanidine from day 6 p.i. resulted in an increased survival of up to 4 days for infected mice (Figure 3H), with the percentage of DAF-FM + macrophages decreasing to less than 12% (Figure 3G). Furthermore, increased survival in VLA-4- treated mice correlated with a significant reduction in NO^+^ macrophages in the brain (Figure 3I) at day 7 p.i. Taken together, these data show that inhibition of Ly6C^hi^ monocyte-derived NO, either by reducing the immigration of these cells into the brain, or by inhibiting NO, promotes survival in WNV encephalitis. 

Although the production of other pro-inflammatory mediators is also likely to occur via inflammatory monocyte-derived macrophages, these data provide one potential lethal effector function for pathogenic macrophages that infiltrate into the WNV-infected brain. The production of NO by macrophages is integral to clearance of many invading pathogens and mediates viral clearance in models of HSV-2, *Ectromelia* and *Vaccinia* virus infection [16]. Over-exuberant NO production can be pathogenic, as exemplified by infection by another flavivirus, Murray Valley encephalitis virus, in which experimental inhibition of NO attenuated disease and prolonged survival [15]. During WNV encephalitis, more than 70% of the inflammatory monocyte-derived macrophages in the WNV-infected brain produced NO. Importantly, whereas inhibition of NO with aminoguanidine over the entire course of infection had no effect on disease outcome, inhibiting NO on day 6 p.i. resulted in enhanced survival of WNV-infected mice. This apparently paradoxical effect suggests that NO may be important in the early control of WNV infection, whereas later, the sustained production of NO by large numbers of infiltrating macrophages becomes poorly regulated and thus pathogenic. This is supported by previous macrophage-depletion studies, which show that early prolonged macrophage depletion can result in brain infection in mice infected with a non-encephalogenic WNV strain [19]. It is important to consider that although NOS-2 *in situ* hybridization studies support a role for macrophages in NO production during WNV encephalitis, NO may also be produced by neurons (neuronal NOS; NOS-1) and endothelium (endothelial NOS; NOS-3). Because arginine is required as a substrate for the production of NO by all forms of NOS, and aminoguanidine competes with arginine at all these sites, we cannot determine with certainty that the effect we found after aminoguanidine administration is due to the inhibition of NO within macrophages, endothelium, or neurons. In addition, in the brain, NO modulates neurotransmission. Because WNV infection of the brain causes severe seizures, and other studies have implicated NO in the modulation of neuronal function [7], the ability of NO inhibition at day 6 p.i. to enhance survival of infected mice may also be the result of aminoguanidine interactions that stabilize neurons within the WNV-infected brain. The lack of seizures seen in interferon- γ (IFN-γ^−/−^) mice, as previously described [7], supports this hypothesis. This is because mice deficient in IFN-γ have reduced ability to produce NO and do not develop seizures [7]. However, in IFN-γ^−/−^ mice, only the NO produced via NOS-2 is reduced. Similar survival kinetics were reported with WNV-infected IFN-γ^−/−^ mice [7] compared with wild-type mice receiving aminoguanidine from day −1 p.i. throughout the disease course. Together this strongly suggests that the antiviral effects of NOS-2-induced NO are important early during infection and contrasts with its evident pathogenic function, probably via similar mechanisms, late in the disease.

In conclusion, we show here that macrophages derived from Ly6C^hi^ inflammatory monocytes predominantly utilize VLA-4 to enter the CNS. Abrogation of this migration by integrin blockade resulted in long-term survival and immunity in an otherwise lethal model. Finally, these data show the crucial contribution by inflammatory macrophages, within in a narrow, temporally defined window, to the immunopathogenesis of disease. This should inform interventional approaches that can ameliorate immunopathology by targeting a specific myeloid subset(s) in the short term, while enabling virus clearance and host survival.

## Abbreviations

APC: Antigen presenting cell; CNS: Central nervous system; CCL: Chemokine ligand; CD: Cluster of differentiation; cFMS-EGFP: colony-stimulating factor 1 receptor-enhanced green fluorescent protein (Mac Green mouse); DAF-FM: 4-amino-5-methylamino-2’,7’-diflourofluorescein diacetate; ICAM: Intercellular adhesion molecule; iNOS: Inducible nitric oxide synthase; LFA-1: Lymphocyte function associated antigen-1; MTT: 3-(4,5-dimethylthiazol-2-yl)-2,5-diphenyltetrazolium bromide; NO: Nitric oxide; PFU: Plaque-forming units; VCAM: vascular cell adhesion molecule; VLA-4: Very late antigen-4; WNV: West Nile virus.

## Competing interests

The authors have no competing interest to report.

## Authors’ contributions

DG & NJC designed infection experiments, performed flow cytometry, immunohistochemistry and drafted the manuscript. MT, RT & CD assisted with experimental design, flow cytomtry and antibody/drug treatments. TMA & JR, optimized and performed immunohistochemistry. MM & MH designed and completed *in-situ* hybridizations experiments. ILC designed *in-situ* primers and assisted with manuscript drafting. All authors read and approved the final manuscript.
